# Exerting pulling forces in fluids by directional disassembly of microcrystalline fibres

**DOI:** 10.1038/s41565-024-01742-x

**Published:** 2024-07-29

**Authors:** L. C. Pantaleone, E. Calicchia, J. Martinelli, M. C. A. Stuart, Y. Y. Lopatina, W. R. Browne, G. Portale, K. M. Tych, T. Kudernac

**Affiliations:** 1 Stratingh Institute for Chemistry, University of Groningen, Groningen, Netherlands; 2 Groningen Research Institute of Pharmacy, University of Groningen, Groningen, Netherlands; 3 Zernike Institute for Advanced Materials, University of Groningen, Groningen, Netherlands; 4 Groningen Biomolecular Sciences and Biotechnology Institute, University of Groningen, Groningen, Netherlands; 5 Institute of Physics of the National Academy of Sciences of Ukraine, Kyiv, Ukraine

**Keywords:** Supramolecular chemistry, Supramolecular polymers

## Abstract

Biomolecular polymerization motors are biochemical systems that use supramolecular (de-)polymerization to convert chemical potential into useful mechanical work. With the intent to explore new chemomechanical transduction strategies, here we show a synthetic molecular system that can generate forces via the controlled disassembly of self-organized molecules in a crystal lattice, as they are freely suspended in a fluid. An amphiphilic monomer self-assembles into rigid, high-aspect-ratio microcrystalline fibres. The assembly process is regulated by a coumarin-based pH switching motif. The microfibre crystal morphology determines the monomer reactivity at the interface, resulting in anisotropic etching. This effect exerts a directional pulling force on microscopic beads adsorbed on the crystal surface through weak multivalent interactions. We use optical-tweezers-based force spectroscopy to extract mechanistic insights into this process, quantifying a stall force of 2.3 pN (±0.1 pN) exerted by the ratcheting mechanism produced by the disassembly of the microfibres.

## Abstract (web summary)

Disassembling molecular microcrystalline fibres produce mechanical work by dragging micro objects along their surface via biased diffusion.

## Main

With the tremendous potential of supramolecular polymerization realized over the past decades, the scope of its application is extending into the field of molecular machinery, a niche that—in nature—is occupied by a class of proteins known as cytoskeletal polymerization motors (PMs)^1^. These biomolecular machines convert chemical energy provided by ATP/GTP hydrolysis into mechanical work via self-assembly processes^2^. These machines drive intracellular modulations, morphology changes, motion and other essential tasks for the survival of organisms including chromosomal segregation during mitosis^3^. Both actin filaments and microtubules organize and orient monomers by self-assembly to amplify molecular events across length scales. Despite the recent advances in controlling molecular motion, harvesting this motion in fluids—an environment dominated by viscous forces and Brownian fluctuations—still remains a major challenge in the field of synthetic molecular machines^4,5^. Additional attributes are needed to realize an artificial PM on the level of sophistication of biomolecular PMs, such as regulation, cooperativity, polarization and dissipation of chemical energy^6–9^. However, any self-assembling system with a fuelled processive behaviour and sufficient structural stiffness has the potential to exert pulling or pushing mechanical forces during (de-)polymerization. Microcrystallization has often been regarded as a model for the self-assembly of molecular architectures of reduced dimensionality, where the lack of dynamicity of the crystal’s bulk and the reduced presence of defects represent the main differences between crystalline structures and supramolecular polymers, that is, supramolecular fibres or tubular assemblies. Fewer than a handful of examples demonstrating the conversion of crystallization energy into mechanical work have been reported: the transport of particles pushed by crystalline growth, deformation of lipid compartments or piercing of water droplets^10–12^. These pioneering examples provide tantalizing hints on what can be achieved with microcrystallization, even though forces originate from a purely physical process. By contrast, chemomechanical transduction in natural PMs is fuelled by chemical transformations rather than a concentration gradient or drying. Direct and quantitative measurements of mechanical forces exerted by the (dis)assembly of monomers constitute another challenge in this field^13^. Force spectroscopy techniques, and more specifically the use of optical tweezers (OT), played a pivotal role in building our current understanding of mechanisms like the conformational wave or Brownian ratcheting, observed in natural PMs^14–16^. To the best of our knowledge, an artificial system showing direct evidence of chemomechanical transduction by means of force spectroscopy has not been reported to date. Performing such measurements in chemically fuelled systems is an essential step for characterizing their mechanical behaviour and assessing mechanistic resemblance with their natural analogues.

**Fig. 1:**
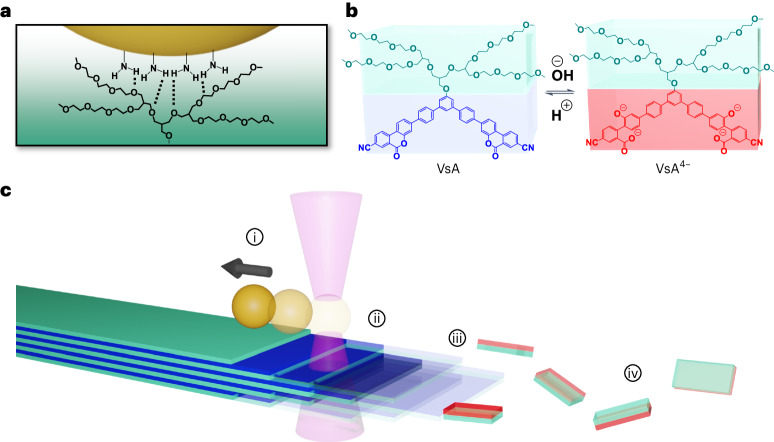
Mechanism of force exertion by disassembling microcrystalline fibres. **a**, Amino-functionalized 1 µm polystyrene beads adsorb onto the crystal surface through a multivalent interaction. **b**, Disassembly of the fibre is controlled by the pH-switchable coumarin motif as part of the aromatic scaffold of the monomer. Under basic pH conditions, the assembled VsA monomer (blue) is hydrolysed into its soluble VsA^4−^ form (red). **c**, Force spectroscopy measurement with OT. During the anisotropic etching of the microcrystals, the beads move by a biased diffusion mechanism. A pulling force (i) displaces the beads from the centre of the optical trap (ii) to maintain the multivalent interaction with the surface. Monomer hydrolysis occurs predominantly at the fibre edge (iii), directing the disassembly process along the fibre axis (iv).

With this in mind, we designed a force spectroscopy experiment where disassembling microcrystalline fibres is used to exert pulling forces on microscopic polystyrene beads multivalently bound to the surface of the crystal (Fig. 1). The crystal morphology imparts directionality to this process: the disassembly is controlled by a pH switching of the monomer whose reactivity is modulated by the surface of the crystal. In fact, the basic digestion of the crystalline material is expected to anisotropically occur, preferentially switching and removing the monomer along the main axis of the fibre. Once the crystal edge reaches the contact area of the bead, according to Hill’s (biased) diffusion model, the bead is pulled in the etching direction to maximize multivalent binding with the surface^17,18^.

## pH switching of the monomer

The monomer used in this study is a V-shaped amphiphile (VsA)^19–21^. Most self-assembled structures responding to pH rely on the deprotonation/protonation of monomer residues^22^. However, compared with these dynamic systems, the benzo[c]coumarin switch in the VsA aromatic scaffold operates through a more complex chemical conversion based on reversible hydrolysis/lactonization. A similar mechanism is often found in chemically fuelled supramolecular polymerizations^23^. Specifically, in those systems where self-assembly is controlled by reactions between carboxylic acids and methylating agents^24,25^ or carbodiimides^26–28^, alkaline conditions are employed to deactivate the resulting esters or anhydride products^29^. Spectroscopic titration of the monomer confirmed a pH actuation range within 5 < pH < 12, consistent with those reported for biaryl lactones with similar donor–acceptor features (Supplementary Fig. 1d)^30^. When the coumarin switch is closed, the monomer is characterized by an absorption band at 320 nm responsible for the observed fluorescence, arising from an intramolecular charge transfer mechanism (Supplementary Fig. 1a,c,e). The hydrolysis of coumarin under basic conditions quenches the fluorescence, and a new absorption band at 346 nm appears, indicating the opening of the switch and the accumulation of tetra anionic monomeric species (VsA^4−^). This species is stabilized against intramolecular esterification. On the protonation of phenolates, the dianion species (VsA^2−^) slowly reverts into the closed form and the switching process accelerates after titration of the carboxylate groups. The reversibility of the process was also confirmed by Fourier transform infrared (FTIR) spectroscopy: on basification with sodium hydroxide, the sharp ester carbonyl stretching band at 1,727 cm^−1^ is replaced by two bands at 1,572 and 1,375 cm^−1^, consistent with the formation of a carboxylate sodium salt (Supplementary Fig. 1a). Subsequent acidification reverts the spectrum to its original state, with VsA in its closed form (Supplementary Fig. 1a).

## Characterization of hierarchically assembled microcrystalline fibres

**Fig. 2:**
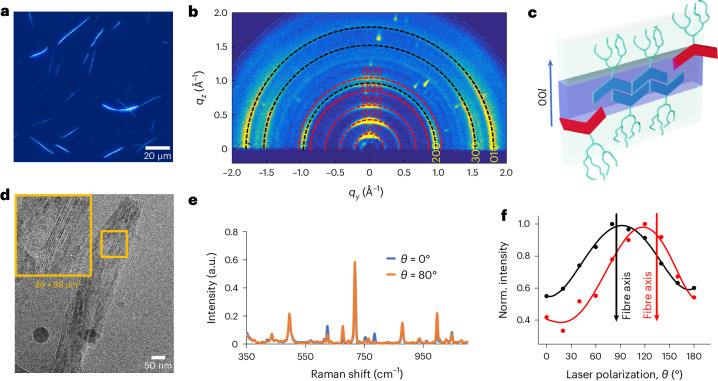
Structural characterization of VsA microcrystalline fibres. **a**, Polarized optical microscopy of dispersed fibres. **b**, GIWAXS diffraction pattern of a thin film of crystalline fibres. **c**, Representation of crystal packing and anisotropic etching at the crystal interface: assembled VsA monomers (blue) are hydrolysed in their soluble VsA^4−^ form (red) and disassembled along the staggering direction. **d**, Cryo-EM micrograph of a crystalline fibre. **e**, Effect of orthogonal polarizations on the Raman spectra of a single-crystalline fibre. **f**, Normalized intensity of Raman scattering (*υ*_2_ = 998 cm^−1^) as a function of laser polarization; the black and red arrows indicate the orientation of the corresponding fibre axis.

The formation of microcrystals was achieved by the spontaneous self-assembly of VsA, induced by a solvent processing method^31^. The monomer was molecularly dissolved in acetonitrile, the solution was dispersed in water (water/ACN = 80/20) and the samples were annealed at 60 °C. Optical microscopy following overnight annealing showed the formation of needle-shaped fluorescent fibres that reach up to tens of micrometres in length. Fibres imaged under cross-polarizers showed birefringence, indicating a crystalline state (Fig. 2a).

The assembly pathway was elucidated by transmission electron microscopy (TEM) and dynamic light scattering investigations. In the early stages of assembly, the VsA forms transient nanofibres with a diameter of *d* = 4.1 nm (±0.2 nm) (Supplementary Fig. 2a). The size of these nanofibres suggests that the amphiphiles pair their aromatic cores to form dimer units that π–π stack on top of each other^32^. At the later assembly stage, the polar environment promotes further association. Here a stratified material is formed on the association of nanofibres into large crystalline domains, which grow up to a micrometre in length within 30 min (Supplementary Fig. 2d,e) and turn into rigid fibres with a high persistence length (*l* = 370 µm ± 96 µm) and tend to fracture under mechanical stress (Supplementary Fig. 2b,c,f,g). Due to their stiffness and anisotropic properties, the latter form of assemblies—microcrystalline fibres—are the main object of this study, and only this type of aggregation state will be used to exert directional pulling forces.

Composition and molecular alignment in the fibres were explored by polarized Raman microspectroscopy. Single-crystalline fibres were sampled with sub-micrometre spatial resolution. The presence of the ester carbonyl stretching band (*υ*_1_ = 1,725 cm^−1^), in line with the FTIR spectra (Supplementary Figs. 1a and 3c), confirmed that under the aforementioned assembling conditions, VsA crystallizes in its closed form. The assignment of vibrational frequencies was based on density functional theory (DFT) calculations. Among the three conformers considered in the DFT study, the simulated spectra of a symmetric VsA isomer with internal lactone rings best approximated the experimental spectra (Supplementary Fig. 3c). The spectra were recorded by varying the orientation of the laser polarization with respect to the long axis of the fibres (Fig. 2e). The oscillating intensity of the central ring breathing band (*υ*_2_ = 998 cm^−1^) was maximized when the polarization was aligned with the fibres (Fig. 2f). Other vibrations oscillated fully out of phase (*υ*_3_ = 624 cm^−1^; *υ*_4_ = 784 cm^−1^) or were unaffected by the polarization of the laser (*υ*_5_ = 496 cm^−1^) (Supplementary Fig. 3b)^33^. These anisotropy effects, originating from the vibrational symmetry and molecular orientation, are consistent with the molecular alignment in the specimen, which persists across the domain of a single fibre^34^.

Grazing-incidence wide-angle X-ray scattering (GIWAXS) analysis was performed on a thin film of fibres drop cast onto a silicon substrate, which further elucidated the molecular packing of VsA inside the fibres (Fig. 2b). The GIWAXS pattern exhibits several diffraction rings, confirming the crystalline nature of the aggregates. The rings present some anisotropy, suggesting a small—but measurable—preferential orientation in the deposition of fibres. Specifically, five intense (00*l*) reflections show a marked directionality, being more intense along the vertical direction. The presence of (00*l*) diffraction signals, where *l* = 1–5, is due to a layer-like structure with periodicity *d*_001 _= 2π/*q*_001_ = 2.8 nm, which we attribute to alternate regions of the hydrophobic aromatic component of the molecule and hydrophilic-glycol-based part of the molecules (Fig. 2c). A similar interplanar distance is observed in cryogenic electron microscopy (cryo-EM) micrographs. Figure 2d shows the regular spacing of stacked planes in the microcrystalline fibres with a periodicity of 2.7 nm (±0.3 nm). The alternating phase contrast due to the difference in the scattering factor is consistent with the layered architecture formed on the entangling of the dendron’s tails.

The GIWAXS pattern features three more clearly visible signals orthogonally oriented with respect to the (00*l*) signals and preferentially located along the horizontal *q*_*y*_ direction, with position *q*_*y*_ = 1.0, 1.5 and 1.8 Å^−1^. The first two signals are ascribed to the diffraction plane generated by the regular staggering along the aromatic backbone, whereas the last one is assigned to the stacking along the π−π direction (Fig. 2b,c). X-ray diffraction (XRD) data from a thin film of fibres deposited on a silicon wafer were used to confirm the structural model and refine the assignment of the unit cell (Supplementary Fig. 4c). By integrating the XRD and GIWAXS data, we derived a unit cell with axis *a* = 12.44 Å, *b* = 3.50 Å and *c* = 28.57 Å for the VsA crystalline fibres.

## Directional disassembly by anisotropic etching

**Fig. 3:**
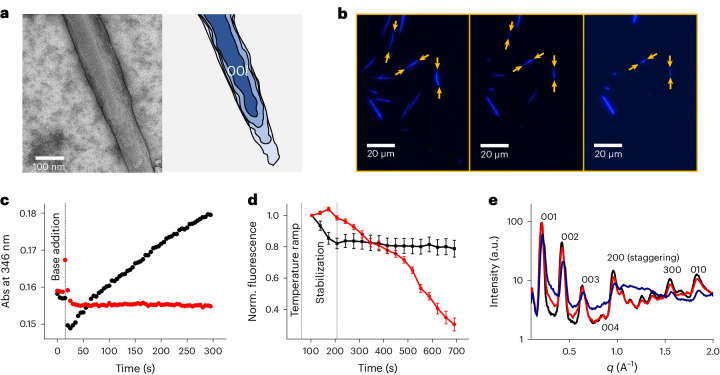
Evidence for anisotropic etching of microcrystalline fibres. **a**, TEM micrograph of a single-crystalline fibre (left) and its representation (right), highlighting the morphology of the 00*l* crystal facet. **b**, Fluorescence microscopy: disassembly of microcrystals during basic hydrolysis (NaOH at 60 °C); micrographs time frame (from left to right): 3 min, 6 min, 13 min. **c**, Ultraviolet–visible absorption spectroscopy: coumarin hydrolysis rate in microcrystalline fibre (red) and nanofibre (black) solutions (NaOH at 20 °C). **d**, Normalized fluorescence intensity of micrographs before (black) and after (red) NaOH addition (60 °C). Data points represent the averaged mean grey values from five ROI; the error bars represent standard deviation. **e**, Radially integrated GIWAXS profiles of partially digested fibres. Thin film of crystalline fibres after deposition (black), after first basic treatment (1 M NaOH at 45 °C for 5 min, water washed) (red) and after second treatment (1 M NaOH at 45 °C for 10 min, water washed) (blue).

The basic hydrolysis of the coumarin switch induces changes in the geometry and polarity of the monomer, resulting in the disassembly of the pre-formed crystalline fibres. The reversibility of this process is limited by the wide operative pH range of the coumarin switch: the switching cycle generates substantial amounts of salt that affect the re-assembly of the fibres resulting in a larger number of small fibres and an increased tendency to flocculation (Supplementary Fig. 4d). The rate of hydrolysis of the monomer critically depends on the state of its assembly. In fresh samples, dominated by the presence of nanofibres, on base addition, the monomer switches into the open VsA^4−^ form at room temperature (Fig. 3c). By contrast, after the microcrystallization of VsA took place, basic hydrolysis required heating the solutions above 30 °C to yield measurable conversion (Fig. 3c). We hypothesize that in the crystal bulk, the monomer is stabilized and due to the lack of exchange with the solvent, the monomer is inaccessible to solvolysis.

In a hierarchically organized highly anisotropic material, different facets of the same crystal are expected to show different reactivities^35,36^. Analysis of the fluorescence microscopy images shows that the crystalline material is preferentially etched from the ends of the fibres, which results in a directional disassembly along the main axis (Fig. 3b) (Supplementary Video 1). Further, the crystals were heated at 60 °C in the absence of the base to exclude the fact that this effect was only caused by a difference in solubility between the crystal facets. Comparing the changes in normalized fluorescence intensity from individual fibres before and after the addition of the base confirms that disassembly does not occur without the basic hydrolysis of the monomer (Fig. 3d).

The occurrence of anisotropic etching was also confirmed by the X-ray scattering analysis of partially digested samples. After removing residual sodium hydroxide and degraded monomer, the radially integrated GIWAXS profiles show an increase in the background caused by the general degradation of the sample (Fig. 3e). Nevertheless, etching is substantially more pronounced on certain reflections, specifically visible for the (*h*00) ‘staggering’ direction. The amorphization of (00*l*) planes remains limited, which is explained by the crystal morphology since in the corresponding facets, the monomers expose their glycol tail to the surface, shielding the coumarin motif from the solvent (Fig. 2c).

## Exerting pulling forces in fluids by directional disassembly

**Fig. 4:**
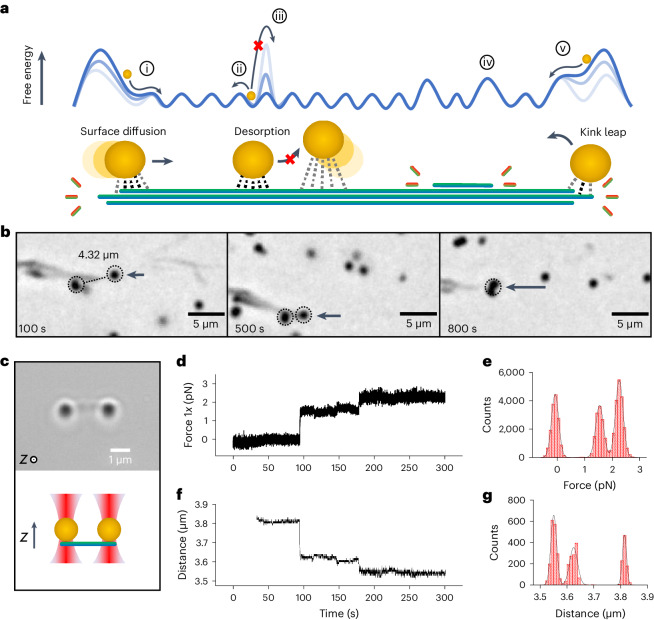
Pulling experiments and force spectroscopy measurements. **a**, Schematic correlating the dynamics of a multivalently bound cargo with a free-energy landscape, showing the effect of anisotropic etching on surface diffusion. Biased (i) and thermal (ii) diffusion, desorption (iii) and effect of asperities caused by crystal morphology on corrugation height ((iv) and (v)). **b**, Pulling motion in a connected pair of beads was observed with microscopy during fibre disassembly. **c**, Bright-field microscopy of beads connected by a bridging fibre in the OT setup. A side view of the setup with the OT configuration is outlined below the micrograph. **d**,**e**, Pulling force generated by the disassembly of a fibre was measured using two optically trapped beads (1 µm). The spring constant of the trap is *k* = 18.3 fN nm^–1^, and the sampling frequency of the force is 150 Hz. **f**,**g**, Sampling frequency of the inter-bead distance is 15 Hz. Work done during the pulling events (*W* = *F*_1*x*_Δ*x*) was calculated assuming the beads experience anticorrelated forces (*F*_1*x*_ = −*F*_2*x*_).

To explore whether the shortening of fibres can effectively exert mechanical pulling forces, the fibres were decorated with multivalently bound microscopic cargo. Specifically, amino-functionalized polystyrene beads were chosen to adsorb onto the crystal surface by hydrogen bonding with the dendron tails of the glycol-rich monomers^37^. These interactions result in the strong adhesion of beads to the surface of the fibres and allow the incorporation of beads in the fibre network after the pre-incubation of the former with the assembling monomer or to decorate individual fibres with beads using the OT as micro-manipulators (Supplementary Fig. 5c–e,g–i). A strong but dynamic multivalent interaction is required to prevent the detachment of cargo, as well as allow its surface diffusion^38^. As shown in Fig. 4a, the latter process implies a lower activation energy due to the simultaneous loosening and formation of dynamic fibre-bead contacts. By contrast, atomic force microscopy (AFM) measurements confirmed that the desorption of microspheres from the crystal surface only occurs on the application of large forces, in the range of nanonewtons (Supplementary Fig. 6a,b). Despite strong adsorption in biased diffusion models, the dynamic nature of the contacts implies that the cargo is capable of randomly exploring the fibre surface by thermal activation, although diffusion on a scale relevant to optical microscopy only occurs under the drive of fibre disassembly (Supplementary Videos 2 and 3)^15,17^. Etching of the fibre networks was first imaged with bright-field and epifluorescence microscopy (Supplementary Videos 4 and 5). Directional motion was detected when beads were interconnected by disassembling fibres, pulling the cargo over micrometre distances (Fig. 4b).

OT force measurements were employed to gain a more quantitative insight into the directional forces exerted during anisotropic etching. This pulling experiment was performed by trapping a pair of beads connected by a bridging fibre (Fig. 4c) (Supplementary Video 6). Following the successful trapping of the pair of beads in two laser beams, the hydrolysis of the fibre was initiated. Force traces corresponding to the individual beads were perfectly anticorrelated, as expected from the two trapped objects connected by a rigid element. As the crystal edge reached the contact area, the distance between the beads decreased. The corresponding displacement from the trap centre was confirmed by a proportional increase in the *F*_*x*_ force traces, whereas notable fluctuations of force were not detected along its orthogonal component (Fig. 4d–g). The beads were displaced until the system reached a stall force of 2.3 pN (±0.1 pN), producing an estimated mechanical work of 85 kT. These figures are in the same range of tension (0.5–3.0 pN) supported by microtubules during in vitro motility assays^11^. The fact that the movement observed was associated with concerted pulling events spanning hundreds of nanometres (shown by the distribution of histograms; Fig. 4e,g) suggests that crystal kinks, which possess a similar spacing according to AFM topography of the crystal edge (Supplementary Fig. 6c,d), may play a role in limiting diffusion along the fibre surface generating sliding or rolling friction^39^. These kinks, with a step size between 10 and 30 nm of height, constitute the main element of asperity of a relatively flat terracing morphology. Therefore, based on how work is supplied according to the force traces, one could speculate that these elements of surface morphology may affect the shape of the energy landscape in the same way corrugation height is considered in the biased diffusion model of kinetochores (Fig. 4a)^15,40^.

Comparing optical microscopy and force spectroscopy pulling experiments, we observed that the restriction of microbead motion has implications on the extent of the disassembly of fibres. In the absence of external forces applied on the beads, the disassembly of the crystal pulls the beads across micrometre distances, fully hydrolysing the fibres at an approximate rate of 200–300 nm min^–1^. In contrast, when the beads are subjected to the force field of the OT, the disassembly initially proceeds at a comparable pace but ceases after reaching the stall force. Because the etching mechanism is halted once the cargo is unable to move and the bead–fibre contact is retained even after 30 min of digestion with the base, we suspect that the polystyrene surface may stabilize the adsorbed monomers in the contact area^41,42^. Therefore, if unable to move, the bead is effectively ‘capping’ the fibre edge, which exposes the most reactive crystal facets, and the result is an increased resistance towards hydrolysis (Supplementary Fig. 7a). In support of this hypothesis, coumarin emission from the surface of the beads was detected long after the complete hydrolysis of the fibre networks (Supplementary Video 5); moreover, this stabilization effect in ‘capped’ crystal edges was occasionally observed with optical microscopy, in pulling experiments where adsorbed beads were immobilized by interaction with the glass surface of the chamber (Supplementary Video 7 and Supplementary Fig. 7b,c).

## Conclusions

In conclusion, we demonstrated that the anisotropic etching of microcrystals suspended in a fluid can result in an effective chemomechanical transduction. Although distant from the sophistication of biological depolymerization machinery, the present system is a synthetic example of mechanical work generation in fluids by crystal disassembly, where the equilibration of the system is driven by complex chemical conversion. The generation of mechanical force in our system can be understood by Hill’s diffusion model, which considers the diffusion of multivalently bound cargo in the direction of the disassembling tracks^14^. The measured mechanical force of 2.3 pN is similar to the forces exerted by disassembling cellular microtubules^11^. The active area of the disassembling crystal that solely contributes to the produced mechanical force is its topmost layer. Consequently, self-assembling architectures as thin as a single layer could, in principle, exert equally large mechanical forces and benefit from a more efficient atom economy and energy conversion. The same ratcheting mechanism stands for any supramolecular polymer that can transfer its directionality to a multivalently bound cargo when disassembling. The diversification of monomer reactivity at the crystal interface provides such a mechanism, imprinting the anisotropy of the lattice into the movement of cargo.

Biased diffusion, which we have demonstrated can operate at the mesoscale, represents a promising strategy to develop new methods for active transport and chemical actuators based on fibre disassembly^43^. Further advances in these directions will require exploring nanofabrication techniques to achieve macroscopic fibre alignment^44,45^. Indeed, scaling the anisotropic properties of microcrystals will be crucial, both for investigating cargo diffusion in fibre networks and for developing novel bulk rheological properties emerging from the energy transduction process.

## Methods

Chemicals and solvents were obtained from commercial sources and used without further purification, unless stated otherwise. Synthesis and characterization of the VsA monomer are reported in the Supplementary Information along with detailed procedures for the sample preparation of VsA microcrystalline fibres.

### Ultraviolet–visible absorption spectroscopy

The spectra were recorded on an Agilent Technologies 8453 ultraviolet–visible spectrophotometer. During the titration experiment, the monomer was initially dissolved in a solution of 1 M sodium hydroxide, which was gradually neutralized with hydrochloric acid solutions at different concentrations (1 M, 100 mM, 10 mM and 1 mM solutions) ([VsA] = 2.5 μM, water). The pH was read in between the spectroscopic measurements using a Mettler Toledo FiveEasy F-20 benchtop pH/mV meter equipped with a pH electrode inLab microprobe.

### FTIR spectroscopy

Infrared spectra were recorded on an Agilent Cary 630 FTIR spectrometer. VsA and Na_4_VsA solid samples were directly deposited on the crystal after drying the samples.

### Fluorescence spectroscopy

Fluorescence excitation and emission of the VsA monomer were recorded with an FS5 spectrofluorometer from Edinburgh Instruments. Here *λ*_exc_ = 320 nm and *λ*_em_ = 436 nm ([VsA] = 250 nM, water, pH = 4.5).

### Fluorescence and cross-polarized optical microscopy

Imaging with fluorescence microscopy was performed in a Nikon ECLIPSE LV100N POL microscope using filters of the following excitation and emission wavelength cut-offs: *λ*_exc_ = 330–380 nm and *λ*_em_ = 420 nm for imaging the VsA fibres; *λ*_exc_ = 510–560 nm and *λ*_em_ = 580 nm for imaging the polystyrene microspheres labelled with fluorescent red. In the disassembly experiments, in the presence of the base, the normalized fluorescence intensity and distance measurements were calculated by analysing the micrographs with the processing package Fiji (ImageJ v.2.14.0/1.54f). For the control experiments on temperature (Fig. 3d), the fluorescence microscopy experiment was carried out under constant irradiation power; five regions of interest (ROI) were selected from the micrographs where fibres were equilibrated on a two-dimensional surface during the whole footage (Supplementary Fig. 4e–h). After background subtraction, the mean grey values from each ROI were normalized and the averaged values were plotted against time. The error bars represent the standard deviation for each timepoint.

### Dynamic light scattering

Dynamic light scattering measurements were performed with a Zetasizer Ultra Red (ZSU3305) from Malvern Panalytical.

### TEM and cryo-EM

TEM images were recorded with a CM120 instrument at 120 keV acceleration voltage, and the samples were prepared using carbon-coated copper grids. Cryo-EM images were recorded with a Tecnai T20 TEM microscope (FEI) operating at 200 keV using a Gatan model 626 cryo-stage sample holder. The distance measurements were performed by analysing the micrographs with the processing package Fiji.

### XRD

Thin-film XRD measurements were carried out using a Bruker D8 Advance diffractometer equipped with a Cu Kα source. The cell parameters were extracted from the diffraction pattern using N-TREOR09 from EXPO2014 software^46^.

### GIWAXS

GIWAXS measurements were performed at the Multipurpose Instrument for Nanostructure Analysis, University of Groningen. This instrument is built on a Cu rotating anode source providing an X-ray beam with a wavelength of *l* = 0.15413 nm. Two-dimensional patterns were collected using a VÅNTEC-500 detector (1,024 × 1,024 pixel array with a pixel size of 136 × 136 μm located 89 mm away from the sample for GIWAXS). The samples were placed in reflection geometry at 0.4° incident angle (*α*) with respect to the direct beam using a Huber goniometer. The direct-beam centre position on the detector and the sample–detector distance were calibrated using the diffraction rings from standard silver behenate and α-Al_2_O_3_ powders.

### Raman microspectroscopy

Solid-state Raman spectroscopy at 785 nm was performed with an Olympus BX51M microscope equipped with a fibre-coupled laser (BT785, ONDAX, 500 mW), a fibre-coupled Andor Shamrock SR-163 spectrograph and an Andor iVac 316 DR-316B-LDC-DD charge-coupled device camera. Polarized Raman microspectroscopy were obtained at 785 nm using ×50 long-working-distance objective on a BX-51 microscope. Excitation was provided by an ONDAX LM-785 free-space laser (75 mW at source), which was passed through a laser-line clean-up filter (Semrock LL01-785), a *λ*/2 retarder and polarizing beamsplitter to control power followed by a second *λ*/2 retarder to control the polarization. The laser was combined with the optical path of the spectrometer with a dichroic mirror (45°) (Semrock Di02-R785) and directed to the microscope with gold mirrors. The Raman scattering passed through the dichroic mirror and a Rayleigh line rejection filter (Semrock BLP01-785R) and was focused with a 35-mm-focal-length plano convex lens into an Andor Kymera-193i spectrograph with a 600 lines mm^–1^ grating blazed at 750 nm and Andor iDus-DU416A-LDC-DD charge-coupled device camera. Spectra were acquired with Andor Solis. Spectra were calibrated with polystyrene or cyclohexane (ASTM E1840). The laser spot size was 0.8 ± 0.2 μm (diameter), estimated with an Edmund Optics 240 lines mm^–1^ ruler as the calibrant.

### AFM

Topography imaging and AFM-based force spectroscopy were performed in air on an Agilent PicoLE system. Images of microcrystalline fibres were obtained in the a.c. mode using standard silicon nitride probes (PPP-NCHR, NANOSENSORS) with a resonance frequency of ~330 kHz. The error in measuring the height was within 6%. Data levelling and background subtraction were performed with Gwyddion software (v.2.65). The force spectroscopy measurements were carried out using NH_3_-functionalized polystyrene microspherical probes (diameter, *d* = 1.0 μm) with spring constant *k* = 0.03 N m^–1^ (PT.PS.NH3, Novascan). The estimate of adhesion force range was based on 50 spectra acquisitions, conducted with different tips and varying sampling locations.

### OT

Force spectroscopy measurements were performed with OT using a commercial setup (C-Trap) from Lumicks operated in the dual-trap regime. The force was measured by a position-sensitive detector in two dimensions on both beads with an acquisition rate of 78 kHz. The measurement of the inter-bead distance was performed by bright-field optical tracking with a resolution of 10 nm at 15 Hz. The acquisition was controlled by Bluelake software (Lumicks). To analyse and store the force-trace data, the force acquisition frequency was downsampled to 150 Hz using the pylake Python package^47^. The trap stiffness was calibrated using the thermal noise method^48^. The force resolution of this calibration was estimated to be 100.0 fN at 150 Hz, with a theoretical limit of 0.4 fN for a typical trap stiffness of 16.5 fN nm^–1^ at room temperature.

### High-resolution mass spectrometry

High-resolution mass spectrometry was performed using a Thermo LTQ Orbitrap XL device with the electrospray ionization method, or alternatively using a Voyager DE-Pro MALDI-TOF instrument (when matrix-assisted laser desorption is specified) as the ionization method. Super dihydroxybenzoic acid was used for matrix preparation.

### Nuclear magnetic resonance

Nuclear magnetic resonance spectra were recorded at room temperature on a Varian 400 or Agilent 400 spectrometer and were referenced against the residual non-deuterated solvent signal.

### DFT

All the calculations were carried out in the ORCA software^49^. Three isomers were optimized, corresponding to two symmetrical conformers and an asymmetrical one. Initial geometries were constructed in the Chemcraft software (v.1.8). The PEG moiety was replaced by a methoxy group for each structure. All calculations were carried out at the B3LYP(G)/def2-SVP level of theory^50–54^. The D3 dispersion correction with Becke–Johnson dumping was applied to account for geometrical dispersion^55,56^. The RIJCOSX approximation, in conjunction with the def2-J auxiliary basis set, was used to speed up integral evaluation^57,58^. The exchange integral grid grid4 was used for all the calculations. Increased accuracy was requested via the NoFinalGrid TightOpt VeryTightSCF keywords. For frequency calculation, Hessian was calculated using numerical integration via the NumFreq keyword.

## Online content

Any methods, additional references, Nature Portfolio reporting summaries, source data, extended data, supplementary information, acknowledgements, peer review information; details of author contributions and competing interests; and statements of data and code availability are available at 10.1038/s41565-024-01742-x.

## Supplementary information

Supplementary Information Supplementary Figs. 1–7, methods for sample preparation and synthesis of VsA.

Supplementary Video 1 Anisotropic etching of microcrystalline fibres observed with epifluorescence microscopy (video speed, 100×; scale bar, 50 µm; *λ*_exc_ = 330–380 nm and *λ*_em_ = 420 nm). Disassembly of fibres was induced by the basic hydrolysis of the monomer (NaOH, 60 °C).

Supplementary Video 2 Thermal diffusion of beads in the absence of hydrolysis observed with bright-field microscopy (video speed, 100×; scale bar, 10 µm) (60 °C). This control experiment shows that the thermal diffusion of cargo adsorbed on the crystal surface is very limited compared with biased diffusion. The strength of the adhesion force is also evident as beads transported by the flow often adsorb on the crystal surface.

Supplementary Video 3 Biased diffusion of bead induced by fibre disassembly observed with bright-field microscopy (video speed, 100×; scale bar, 10 µm) (NaOH, 60 °C). During the hydrolysis of fibres, the movement of the adsorbed beads remains minimal if the fibre edge does not reach the contact area.

Supplementary Video 4 Biased diffusion of bead induced by fibre disassembly observed with bright-field microscopy (video speed, 100×; scale bar, 10 µm) (NaOH, 60 °C). In this video, both bead and fibre are visible during the pulling event. The directions of cargo diffusion and crystal etching coincide. By serendipity, the movement observed counters the chamber’s flow.

Supplementary Video 5 Disassembly of fibres exerts enough pulling force to drag a connected triplet of beads close together. Pulling of beads was observed with epifluorescence microscopy (video speed, 100×; beads diameter, 1 µm; *λ*_exc_ = 330–380 nm and *λ*_em_ = 420 nm for imaging fibres; *λ*_exc_ = 510–560 nm and *λ*_em_ = 580 nm for imaging beads). Directional and coupled motion of connected beads were detected during pulling. Disassembly of the fibres was induced by basic hydrolysis of the monomer (NaOH, 35 °C).

Supplementary Video 6 Bright-field microscopy of a pair of beads connected by a bridging fibre in the OT setup. Disassembly of the fibres was induced by the basic hydrolysis of the monomer (NaOH, 35 °C). At the end of the experiment, one of the beads was released from the optical trap to show that the fibre was still connecting the pair.

Supplementary Video 7 Asymmetric etching of fibre axis caused by the stabilization of the crystal edge by an anchored polystyrene bead observed with bright-field microscopy (video speed, 100×) (NaOH, 60 °C).

## Data Availability

The raw data from spectroscopic characterizations, microscopy, X-ray and optical tweezers experiments that support the findings of this study are available via Mendeley data repository NNANO-23123333 V2 at 10.17632/2w44nd59c5.2.
