# Supplementary material for: Exerting pulling forces in fluids by directional disassembly of microcrystalline fibres
Source: Nat Nanotechnol. 2024 Jul 29;19(10):1507–13. doi: 10.1038/s41565-024-01742-x (PMC11486658; doi:10.1038/s41565-024-01742-x)
Supplement: Supplementary file 1 — Supplementary Figs. 1–7, methods for sample preparation and synthesis of VsA. [file 41565_2024_1742_MOESM1_ESM.pdf]

nature nanotechnology

Supplementary information https://doi.org/10.1038/s41565-024-01742-x

Exerting pulling forces in fluids by directional disassembly of microcrystalline fibres

In the format provided by the authors and unedited

Table of contents

1. Methods for sample preparation

1.1 General procedure for preparation of VsA micro-crystalline fibres for optical microscopy…………………………S 2

1.2 General procedure for preparation of VsA micro-crystalline fibres for DLS………………………………………………S 2

1.3 General procedure for preparation of VsA microcrystalline fibres for TEM and Cryo-EM…………………………S 2

1.4 General procedure for preparation of drop casted films for GIWAXS, XRD, Raman and AFM analysis……….S 2

1.5 General procedure for preparation of VsA micro-crystalline fibres for OTs experiment…………………………….S 2

2. Supplementary Figures

2.1 Supplementary Figure 1: pH switch characterization……………………………………………………………………………S 3

2.2 Supplementary Figure 2: Hierarchical assembly timeline………………………………………………………………………S 4

2.3 Supplementary Figure 3: Polarized Raman micro-spectroscopy……………………………………………………………S 5

2.4 Supplementary Figure 4: X-ray diffraction and scattering experiments…………………………………………………S 6

2.5 Supplementary Figure 5: Force Spectroscopy with optical tweezers………………………………………………………S 7

2.6 Supplementary Figure 6: Atomic Force Microscopy…………………………………………………………………………………S 8

2.7 Supplementary Figure 7: Stabilization effect caused by immobilized cargo……………………………………………S 9

3. Synthesis

3.1 Synthesis of methyl 4'-chloro-4-cyano-2'-methoxy-[1,1'-biphenyl]-2-carboxylate (1)……………………………….S 10

3.2 Synthesis of 3-chloro-6-oxo-6H-benzo[c]chromene-8-carbonitrile (2)..S 10

3.3 Synthesis of 6-oxo-3-(4-(trimethylsilyl)phenyl)-6H-benzo[c]chromene-8-carbonitrile (3)...........................S 11

3.4 Synthesis of 3-(4-iodophenyl)-6-oxo-6H-benzo[c]chromene-8-carbonitrile (4)..S 11

3.5 Synthesis of 6-oxo-3-(4-(4,4,5,5-tetramethyl-1,3,2-dioxaborolan-2-yl)phenyl)-6H-benzo[c]chromene-8-carbonitrile (5)..S 12

3.6 Synthesis of dendron precursor (6)………………………………………………………………………………………………………S 13

3.7 Synthesis of VsA monomer...S 13

1. Methods for sample preparation

1.1 General procedure for preparation of VsA micro-crystalline fibres for optical microscopy: a stock solution of monomer in acetonitrile ([VsA]=100 µM) was diluted and dispersed in MilliQ water (water/acetonitrile 80:20) to adjust the final concentration of monomer to 5 µM. The sample was annealed in a thermostated bath at 60°C which was left cooling at room temperature for 10 hours.

1.2 General procedure for preparation of VsA micro-crystalline fibres for DLS: All solvents were previously filtered through a 0.45 µm membrane, a stock solution of monomer in acetonitrile ([VsA]=100 µM) was diluted and dispersed in MilliQ water (water/acetonitrile 80:20) to adjust the final concentration of monomer to 5 µM. The sample was pre-heated at 60°C and measured in a DTS0012 cell thermostated at 20°C after 5 minutes of equilibration.

1.3 General procedure for preparation of VsA microcrystalline fibres for TEM and Cryo-EM: a stock solution of monomer in acetonitrile ([VsA]=100 µM) was diluted and dispersed in MilliQ water (water/acetonitrile 80:20) to adjust the final concentration of monomer to 10 µM. The sample was annealed in a thermostated bath at 60°C which was left cooling at room temperature for 10 h. After ageing the samples for 24 hours, microcrystalline fibres flocculate and the sample can be concentrated up to 100 times by separating the precipitate from the supernatant and re-dispersing the fibres by mechanical agitation. The resulting solutions were cast on the desired surface and blotted shortly after deposition. TEM samples were stained with a 2% PTA solution (pH=7).

1.4 General procedure for preparation of drop casted films for GIWAXS, XRD, Raman and AFM analysis: a stock solution of monomer in acetonitrile ([VsA]=100 µM) was diluted and dispersed in MilliQ water (water/acetonitrile 80:20) to adjust the final concentration of monomer to 10 µM. The sample was annealed in a thermostated bath at 60°C which was left cooling at room temperature for 10 hours. After ageing the samples for 24 hours, microcrystalline fibres flocculate and the sample can be concentrated up to 100 times by separating the precipitate from the supernatant and re-dispersing the fibres by mechanical agitation. The resulting solutions were cast on the desired surface (silicon wafer for GIWAXS, XRD and AFM; aluminium foil for Raman), blotted after deposition and the residual solvent was dried under reduced pressure.

1.5 General procedure for preparation of VsA micro-crystalline fibres for OTs experiment: a stock solution of monomer in acetonitrile ([VsA]=100 µM) was diluted and dispersed in MilliQ water (water/acetonitrile 80:20) to adjust the final concentration of monomer to 5 µM. The sample was annealed in a thermostated bath at 60°C which was left cooling at room temperature for 10 hours. The fibre suspension was loaded in a custom-made microscopy chip with a 40 µL capacity and 0.3 mm thickness. The chip was constituted by three communicating chambers designed to minimize mixing their content by diffusion. After loading the fibre solution in the first compartment, the two other chambers were loaded with a solution of 1M NaOH and a suspension of 1µm amino-functionalized polystyrene microspheres respectively. After immobilizing a single crystalline fibre using two optically trapped microspheres (See Supplementary Fig. 5c), the fibre was moved into the chamber filled with the alkaline solution and the temperature of the objective and condenser was increased to 35°C to induce the hydrolyzation of the fibres. The effect of temperature change on the trap stiffness was assessed by comparison of the spring constant with the value extrapolated from a trap calibration; the offset at 35°C was found negligible (Δk=0.4%) and no correction was applied (See Supplementary Fig. 5e).

2. Supplementary Figures

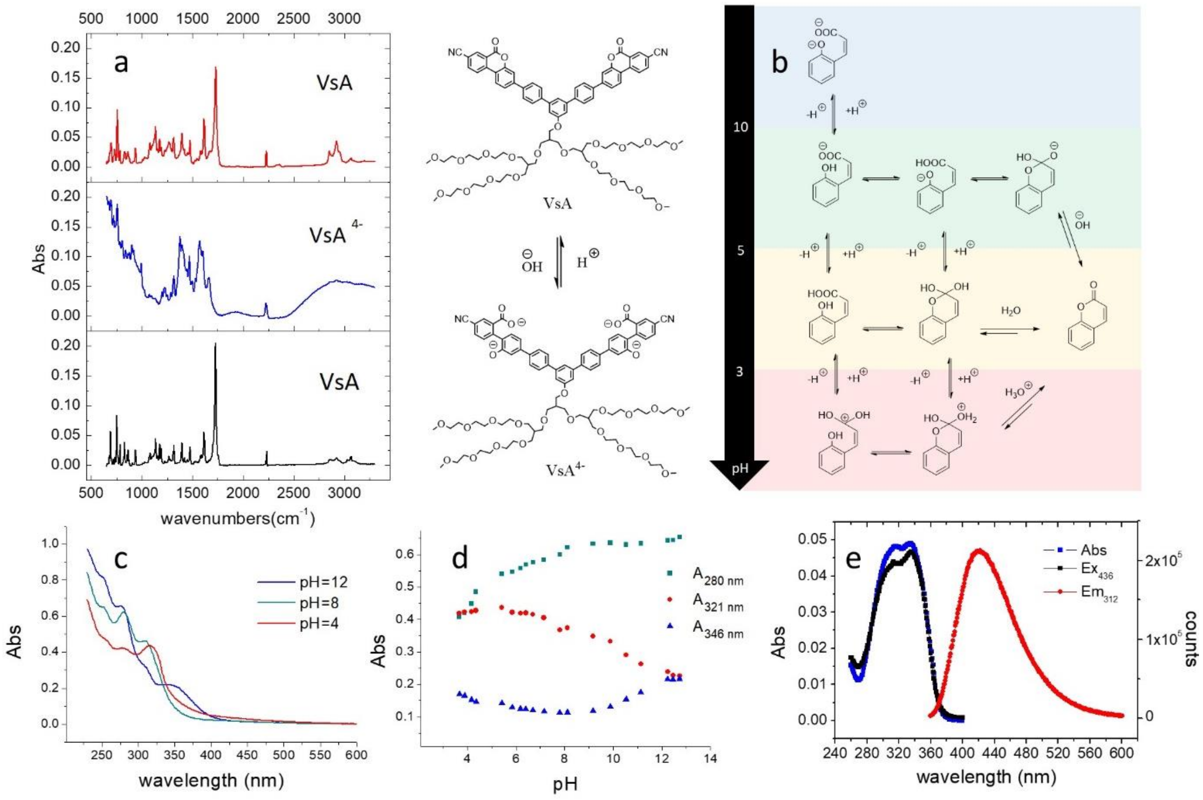

Supplementary Figure 1| pH switch characterization. (**a**) FTIR spectra of VsA monomer (red), VsA^{4-} sodium salt obtained after hydrolysis of the monomer with NaOH (blue), VsA monomer reformed upon neutralization with HCl (black). (**b**) Hypothesized mechanism of hydrolysis/lactonization of a coumarin switch. The donor-acceptor character of the VsA monomer and its poor solubility in water contribute to pushing the equilibrium toward the lactonized form. (**c**)(**d**) Spectroscopic titration of VsA monomer followed by UV-Vis. (**e**) Absorption (blue), excitation (black) and emission spectra (red) of VsA monomer.

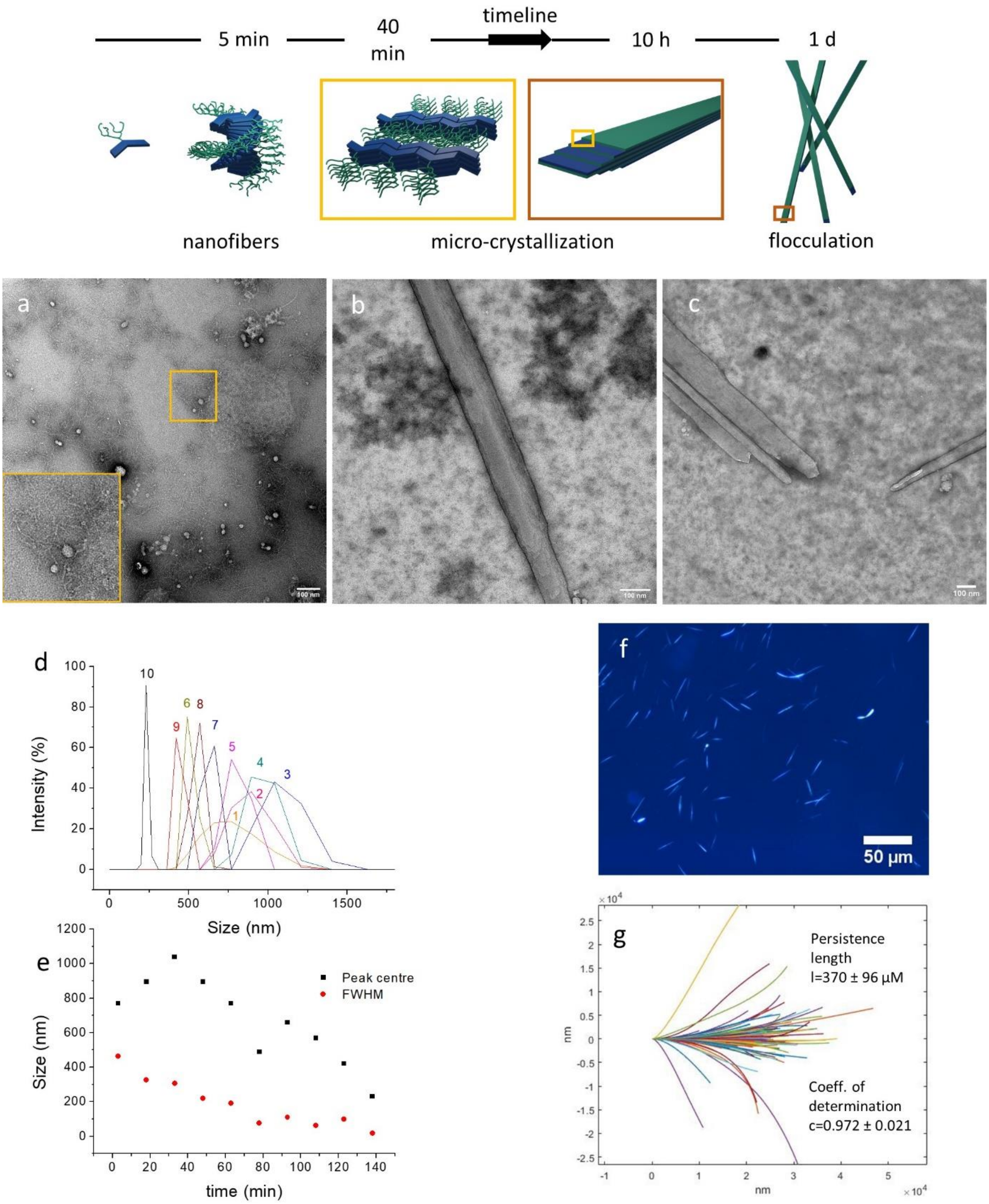

Supplementary Figure 2| Hierarchical assembly timeline. (**a**)Transmission electron microscopy of VsA nanofibers imaged in the early stages of self-assembly. (**b**) Transmission electron microscopy of VsA fibres after micro-crystallization. (**c**) Transmission electron microscopy image of VsA fibres, the crystalline material seems to fracture when subjected to prolonged mechanical stress of sonication (**d**)(**e**) The size and distribution of VsA aggregates were analyzed by dynamic light scattering over time. (**f**)(**g**) Micro crystalline fibers imaged with cross-polarized optical microscopy and corresponding deviation-secant midpoint analysis of the micrograph.

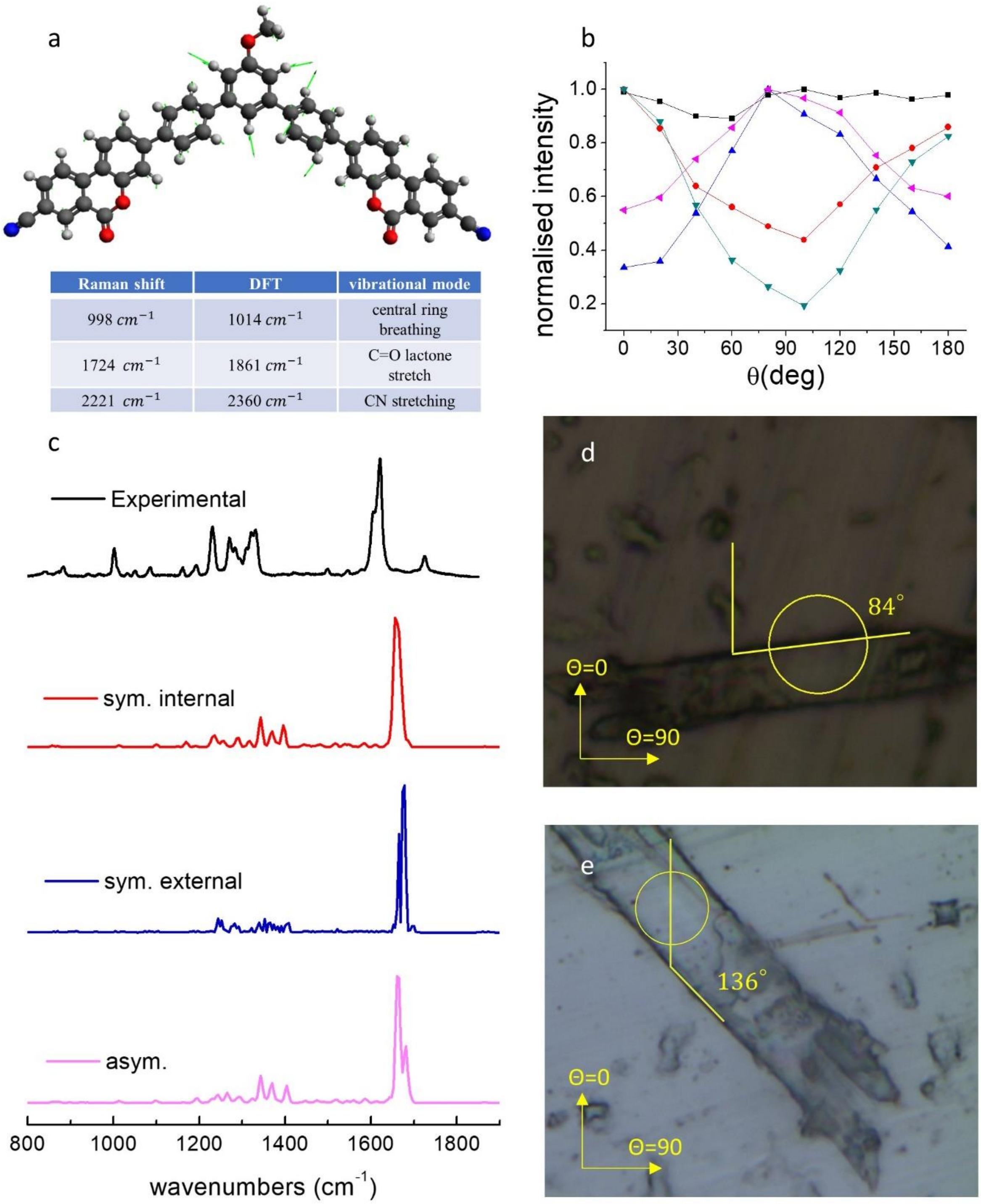

Raman shift	DFT	vibrational mode
998 cm^{-1}	1014 cm^{-1}	central ring breathing
1724 cm^{-1}	1861 cm^{-1}	C=O lactone stretch
2221 cm^{-1}	2360 cm^{-1}	CN stretching

Supplementary Figure 3| Polarized Raman micro-spectroscopy. (**a**) DFT graphic representing the central ring breathing vibration in the symmetrical VSA conformer with internal lactone rings. (**b**) Normalized intensity of Raman scattering for different vibrations as a function of laser polarization (pink, υ_2=998 cm^{-1}; red υ_3=624 cm^{-1}; green, υ_4=784 cm^{-1}; black, υ_5=496cm^{-1}; blue, υ_6=678 cm^{-1}). (**c**) Comparison between experimental Raman spectra of VsA fibres and DFT simulation. Experimental (bulk) spectra was obtained by sampling a region where multiple deposited fibres were superimposed with random orientations to average the fibres' orientation and minimize the effects of polarization. (**d**)(**e**) Micrographs of single crystalline VsA fibres during spectral analysis, estimated laser spot size is 0.8±0.2 µm (yellow circle), fibre orientation is reported with respect to the laser polarization.

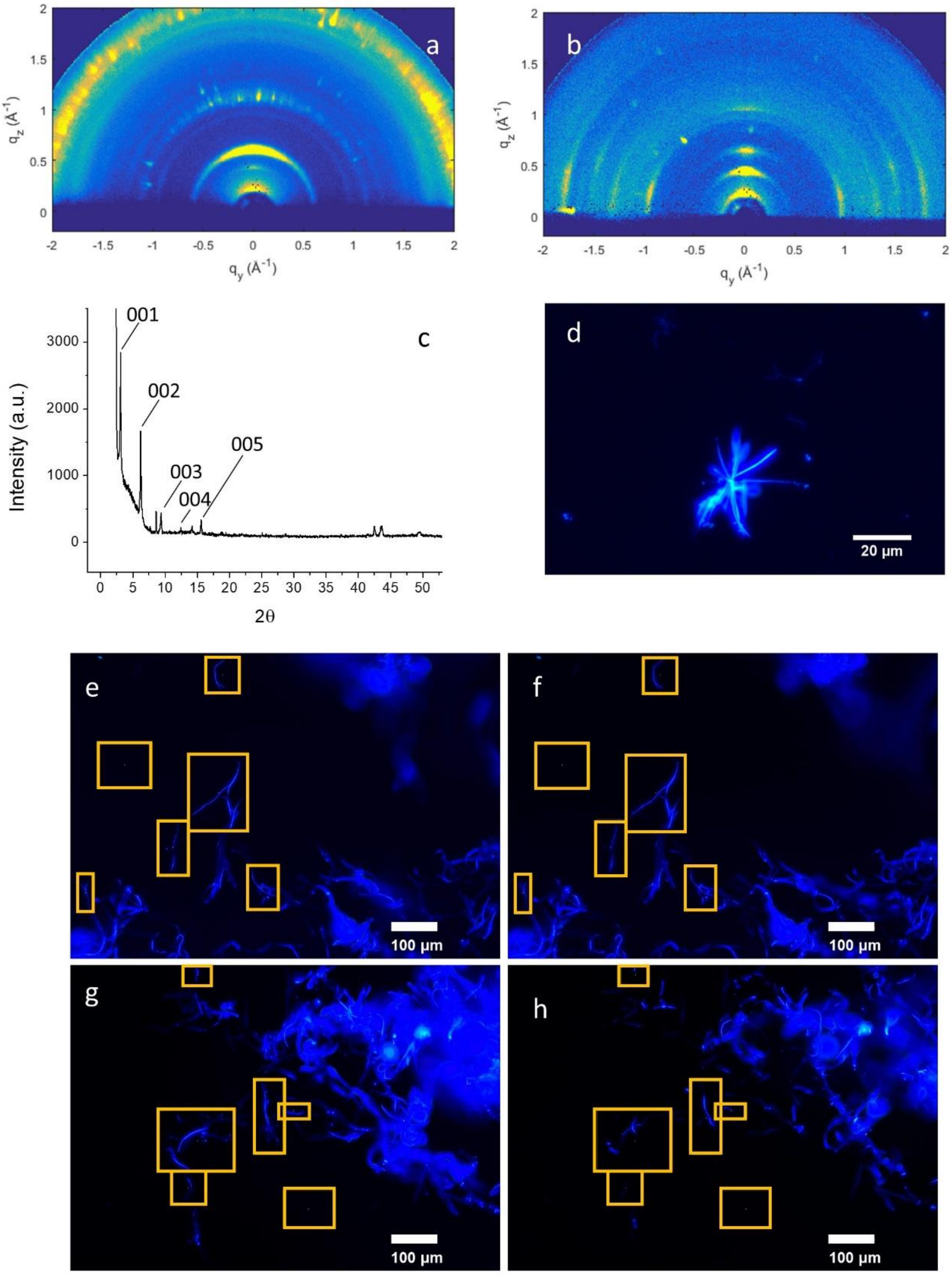

Supplementary Figure 4| X-ray diffraction and scattering experiments. GIWAXS pattern from a partially hydrolyzed sample before (**a**) and after (**b**) washing away the residues of NaOH and digested monomer. (**c**) Diffraction of VsA from a thin film deposition of microcrystalline fibres. (**d**) Fibres formed after neutralization of an alkaline solution of hydrolyzed monomer ([VSA^{4-}]=2.5 µM). The increase in salt concentration caused by the switching cycle ([NaCl]~1mM) influences the self-assembly inducing the formation of smaller fibres and increasing the flocculation of aggregates. (**e**)(**f**) fibre disassembly, control experiment on temperature effect in the absence of base: initial (left) and final (right) frames with selected ROI. (**g**)(**h**) Fibre disassembly, control experiment on temperature effect in the presence of base: initial (left) and final (right) frames with selected ROI.

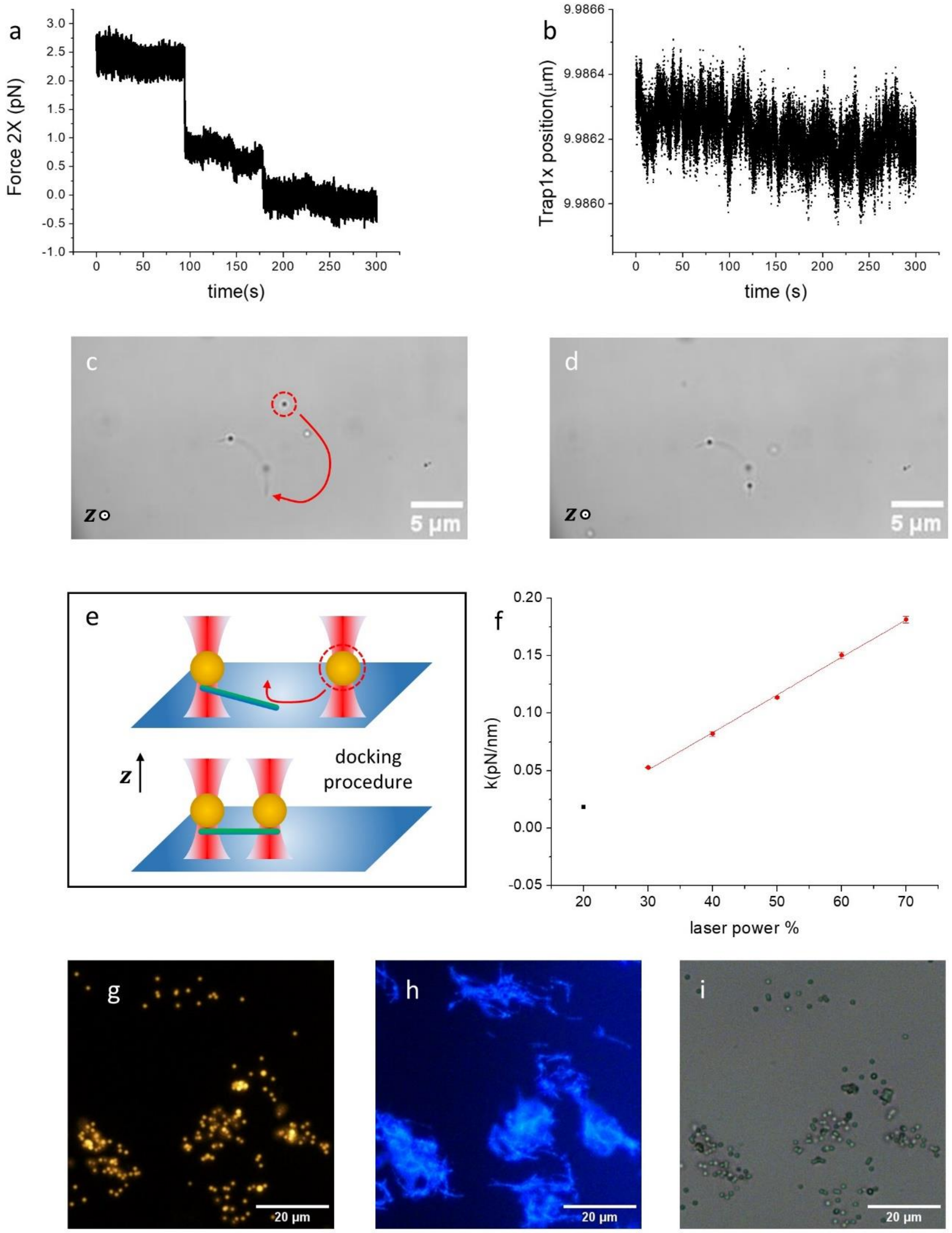

Supplementary Figure 5| Force Spectroscopy with optical tweezers. (**a**) Force measurement from the second trapped bead. The comparison of force traces from the two traps shows that pulling events are anti-correlated, as expected for motions on this timescale for a fibre that is not attached to any other surfaces. (**b**) Position of the trap centre during the pulling experiment. (**c**)(**d**)(**e**) Bead docking procedure for the preparation of a fibre bridge in the OT setup. (**f**) Calibration of spring constant values at 35°C (red, Adj. R-Square 0.99834, Intercept -0.047, Slope 0.00326), spring constant used during force spectroscopy measurements (black, k=18.2665 fN/nm), measured operative laser power P(100%)=14 mW. (**g**)(**h**)(**i**) Fluorescence micrographs and bright field of beads pre-incubated with assembling monomer.

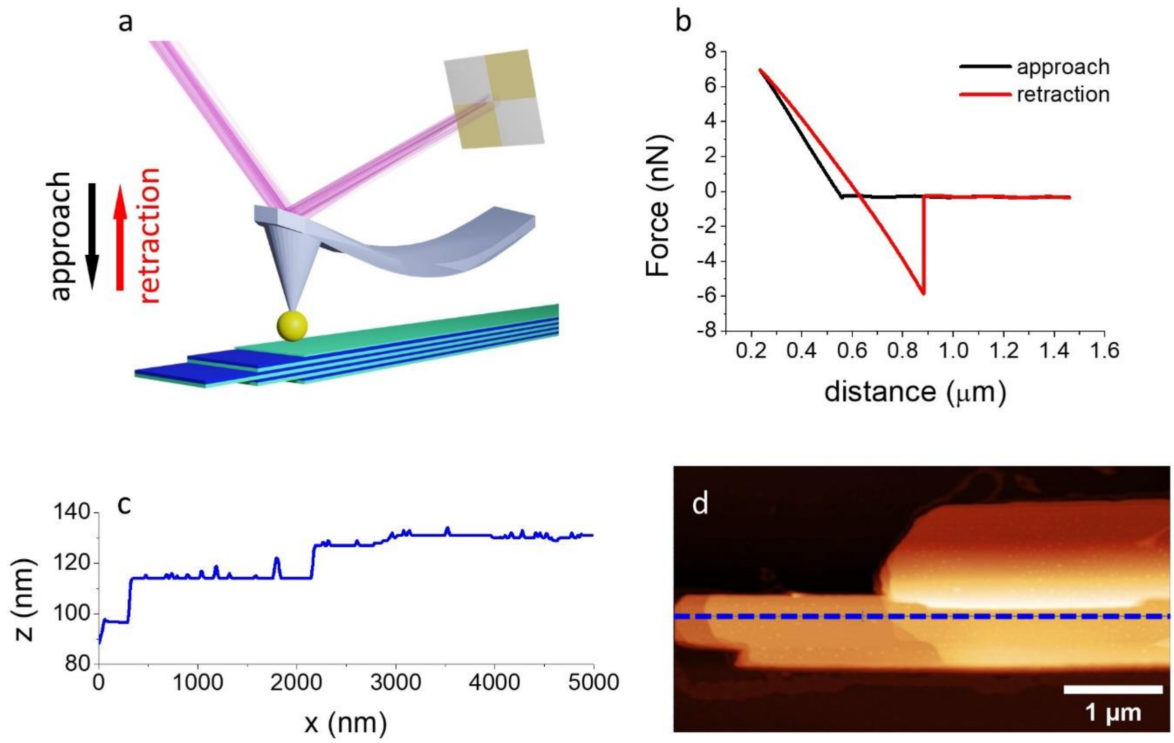

Supplementary Figure 6| Atomic Force Microscopy. (**a**) Representation of the experimental setup for adhesion force measurement between AFM colloidal probe and crystal surface. The cantilever used for this experiment is equipped with a spherical colloidal probe of amino functionalized polystyrene (d=1μm). (**b**) Adhesion force measurement. The force required to desorb the AFM colloidal probe from the crystal surface is 5.85 nN. (**c**) z-profile of the crystal edge and (**d**) corresponding AFM topography imaging, blue dashed line is the plotted profile. Topography reveals that the crystal edge is characterized by a flat terracing morphology, where smooth surfaces extending hundreds of nanometres are spaced by crystal steps of 10-30 nm height.

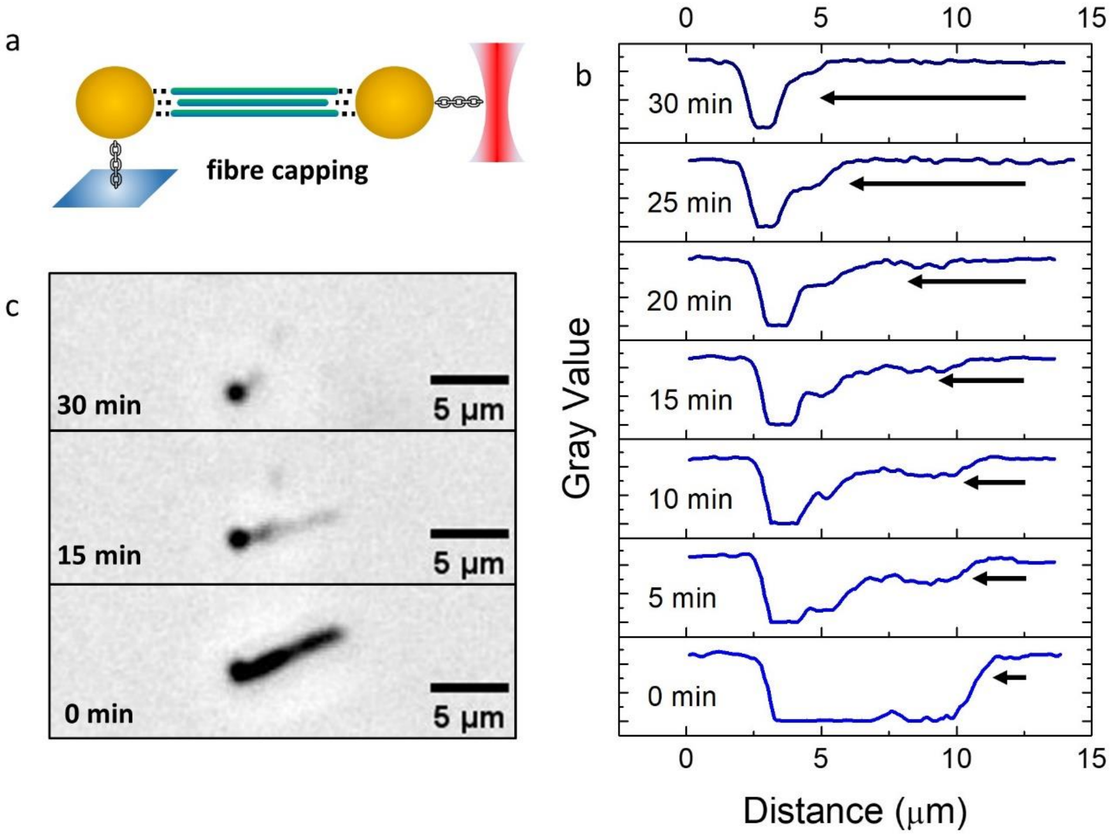

Supplementary Figure 7| Stabilization effect caused by immobilized cargo. (a) Beads can stabilize the fibre edge by adsorbing hydrolysis-sensitive crystal facets. This mechanism holds as long as the cargo is unable to move, immobilized by the force field of the optical tweezers or by a strong interaction with the glass surface. (**b**)(**c**)The latter example was captured in Supplementary Video 7, image analysis from the corresponding micrographs proves that the disassembly along the fibre axis occurs asymmetrically, with the capped end of the crystal showing greater etching resistance.

3. Synthesis

3.1 Synthesis of methyl 4'-chloro-4-cyano-2'-methoxy-[1,1'-biphenyl]-2-carboxylate (1)

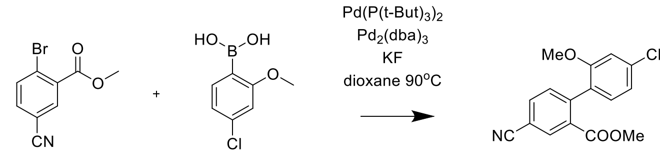

The aryl bromide 2.00 g (8.3 mmol, 1 eq), and KF 1.46 g (24,9 mmol, 3 eq) was added to a Schlenk tube under nitrogen. Next, the boronic acid 1.55 g (8.3 mmol, 1 eq) and 80 mL of dry dioxane were added, followed by a solution of 42 mg of $Pd(P(t\text{-}Bu)_3)_2$ (0.083 mmol, 1%) and 76 mg of $Pd_2(dba)_3$ (0.083 mmol, 1%) in dry dioxane. Three freeze-pump-thaw cycles were performed before the stirring the reaction mixture at 90°C for 48h. Once TLC analysis confirmed the full conversion of the starting material, the reaction mixture was diluted with Et_2O, filtered through a pad of celite with copious washings and concentrated, yielding after purification by column chromatography on silica gel (pentane/diethyl ether 80:20) 1.76 g product **1** as a white solid (yield 70%).

^1H NMR (400 MHz, $CDCl_3$) δ 8.16 (dd, *J* = 1.8, 0.5 Hz, 1H), 7.80 (dd, *J* = 8.0, 1.8 Hz, 1H), 7.41 (dd, *J* = 8.0, 0.5 Hz, 1H), 7.15 (d, *J* = 8.1 Hz, 1H), 7.05 (dd, *J* = 8.1, 1.9 Hz, 1H), 6.91 (d, *J* = 1.9 Hz, 1H), 3.72 (s, 3H), 3.71 (s, 3H)

^{13}C NMR (101 MHz, $CDCl_3$) δ 166.40, 156.37, 142.45, 135.48, 134.62, 133.29, 132.67, 132.23, 130.27, 127.31, 121.10, 117.92, 111.56, 111.24, 55.52, 52.28.

HRMS-ESI Orbitrap (m/z): $[M+H^+]$ calculated for $C_{16}H_{12}ClNO_3H$, 302.05786; found 302.05806

m.p.: 112.1-112.9 °C

3.2 Synthesis of 3-chloro-6-oxo-6H-benzo[c]chromene-8-carbonitrile (2)

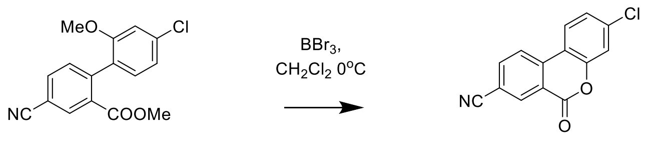

In a round bottom flask 143 mg of compound **1** (0.47 mmol, 1 eq) were dissolved in 5 mL of dry CH_2Cl_2. The reaction flask was chilled at 0°C while purging the solution with nitrogen. After 15 minutes 1.4 mL of 1M BBr_3 solution in CH_2Cl_2 (1.42 mmol, 3 eq) were injected dropwise in the reaction mixture under inert atmosphere. Once the addition was completed the reaction was stirred at 0 °C for 60 minutes. Subsequently, 3.5 mL of cold water were added to the reaction mixture and the organic layer was extracted with 3x5 mL CH_2Cl_2. The combined extracts were washed with 3x20 mL brine solution, dried over Na_2SO_4 and concentrated. The crude was then filtered over a pad of silica, washed with a mixture of pentane/ether (80:20) and eluted with CH_2Cl_2. The resulting solution was concentrated under reduced pressure to afford 85 mg of product **2** a**s** a white solid (yield 71%).

^1H NMR (400 MHz, $CDCl_3$) δ 8.70 (d, *J* = 1.7 Hz, 1H), 8.19 (d, *J* = 8.4 Hz, 1H), 8.10 – 8.04 (m, 1H), 8.01 (d, *J* = 8.5 Hz, 1H), 7.44 (s, 1H), 7.43 – 7.37 (m, 1H).

^{13}C NMR (101 MHz, $CDCl_3$) δ 158.68, 152.17, 138.25, 137.55, 137.18, 135.16, 125.74, 124.50, 122.90, 121.62, 118.45, 117.19, 115.23, 113.02,

MS-MALDI TOF (m/z): $[M^-]$ calculated for $C_{14}H_6ClNO_2$, 255.0087; found 254.9703

m.p.: 311.8-312.7 °C

3.3 Synthesis of 6-oxo-3-(4-(trimethylsilyl)phenyl)-6H-benzo[c]chromene-8-carbonitrile (3)

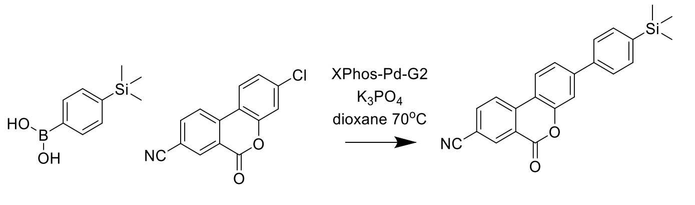

2 3

A three neck flask equipped with reflux condenser was loaded with 11 mg of XPhos-Pd-G2 (0.014 mmol, 3%), 467 mg of $K_3PO_4{\cdot}7H2O$ (1.4 mmol, 3eq), 90 mg of boronic acid (0.69 mmol, 1.5 eq) and dissolved in 8 mL of dry dioxane. The mixture was degassed for 10 minutes under nitrogen before adding 117 mg of **2** (0.46 mmol, 1 eq). The reaction mixture was stirred at 70 °C for 3 hours. The reaction mixture was diluted with 4 mL of ethyl acetate, filtered through a thin pad of celite and concentrated under reduced pressure. The crude obtained was purified via column chromatography on silica gel (CH_2Cl_2/pentane 80:20), yielding 148 mg of product **3** as a white solid (yield 87%).

^{1}H NMR (400 MHz, $CDCl_3$) δ 8.71 (d, *J* = 1.8 Hz, 1H), 8.24 (d, *J* = 8.4 Hz, 1H), 8.13 (d, *J* = 8.3 Hz, 1H), 8.05 (dd, *J* = 8.4, 1.8 Hz, 1H), 7.65 (dp, *J* = 5.4, 1.7 Hz, 6H), 0.32 (s, 9H).

^{13}C NMR (101 MHz, $CDCl_3$) δ 159.37, 152.33, 145.59, 141.52, 138.90, 138.22, 136.98, 135.11, 134.18, 126.35, 123.93, 123.89, 122.87, 121.71, 117.42, 116.11, 115.41, 112.48, -1.17.

HRMS-ESI Orbitrap (m/z): [M+H$^+$] calculated for $C_{23}H_{19}NO_2SiH$, 370.12568; found 370.12826

m.p.: 211.3-213.1 °C

3.4 Synthesis of 3-(4-iodophenyl)-6-oxo-6H-benzo[c]chromene-8-carbonitrile (4)

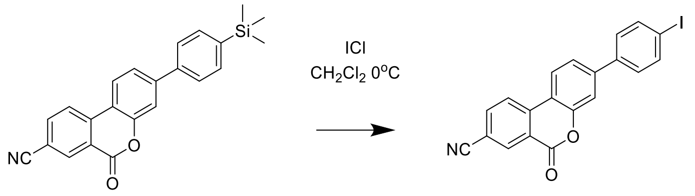

3 4

A round bottom flask was loaded with 154 mg of **3** (0.42 mmol, 1 eq) and dissolved in 15 mL of dry CH_2Cl_2. The flask was placed under nitrogen atmosphere and chilled for 10 minutes at 0 °C before adding dropwise 840 uL of 1 M solution of ICl in CH_2Cl_2 (0.84 mmol, 2 eq). The reaction mixture was kept in the dark, stirring at room temperature overnight. Once the reaction was completed, the reaction mixture was first washed with 30 mL of 0.1 M NaOH and, subsequently, with 3x20 mL of brine. After drying the organic phase with Na_2SO_4, the solvent was evaporated, and the crude was recrystallized by solvent diffusion ($CHCl_3$/Pentane), yielding 169 mg of product **4** as a white solid (yield 95%).

^{1}H NMR (400 MHz, CDC_3) δ 8.72 (d, *J* = 1.6 Hz, 1H), 8.25 (d, *J* = 8.5 Hz, 1H), 8.13 (d, *J* = 8.4 Hz, 1H), 8.06 (dd, *J* = 8.4, 1.8 Hz, 1H), 7.85 (d, *J* = 8.4 Hz, 2H), 7.64 – 7.58 (m, 2H), 7.43 – 7.36 (m, 2H).

^{13}C NMR (101 MHz, $CDCl_3$) δ 159.10, 152.34, 144.36, 138.27, 138.12, 137.96, 136.95, 135.08, 128.74, 124.03, 123.52, 122.83, 121.77, 117.26, 115.93, 115.71, 112.67, 94.83.

HRMS-ESI Orbitrap (m/z): [M+H$^+$] calculated for $C_{20}H_{10}INO_2H$, 423.98290; found 423.98250

m.p.: 300.6-302.1 °C

3.5 Synthesis of 6-oxo-3-(4-(4,4,5,5-tetramethyl-1,3,2-dioxaborolan-2-yl)phenyl)-6H-benzo[c]chromene-8-carbonitrile (5)

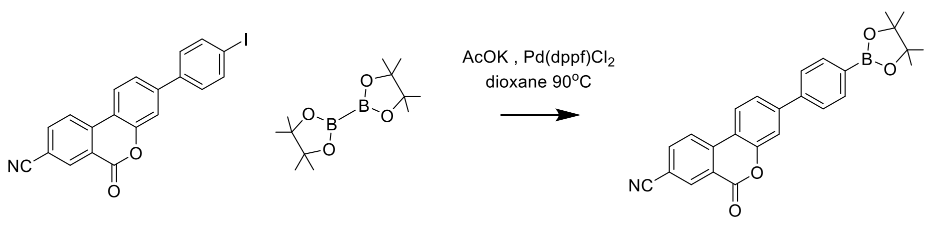

4 5

A three neck flask equipped with reflux condenser was loaded with 168 mg of **4** (0.40 mmol, 1 eq), 119 mg of KOAc (1.20 mmol, 3 eq.), 154 mg of bis(pinacolato)diboron (0.61 mmol, 1.5 eq.) and a catalytic amount of Pd(dppf)Cl_2 (11.7 mg, 16 µmol, 4%) under nitrogen atmosphere. After addition of 15 mL of dry and degassed dioxane, the reaction mixture was stirred at 90˚C overnight. Next, the solution was cooled down, diluted with dichloromethane and washed with 3x15mL of water, then dried over Na_2SO_4, filtered and concentrated. To remove the excess of bis(pinacolato)diboron, the crude was first washed with pentane and subsequently purified by column chromatography on silica gel (CH_2Cl_2/Heptane 80:20) yielding 25 mg of product **5** as a white solid (yield 15 %).

^{1}H NMR (400 MHz, $CDCl_3$) δ 8.70 (d, *J* = 1.7 Hz, 1H), 8.24 (d, *J* = 8.4 Hz, 1H), 8.12 (d, *J* = 8.3 Hz, 1H), 8.04 (dd, *J* = 8.4, 1.8 Hz, 1H), 7.94 (d, *J* = 8.2 Hz, 2H), 7.69 – 7.63 (m, 4H), 1.37 (s, 12H).

^{13}C NMR (101 MHz, $CDCl_3$) δ 159.30, 152.33, 145.44, 141.17, 138.17, 136.98, 135.57, 135.13, 126.37, 123.99, 123.91, 122.91, 121.79, 117.41, 116.31, 115.61, 112.55, 84.04, 24.88.

HRMS-ESI Orbitrap (m/z): [M+H$^+$] calculated for $C_{26}H_{22}BNO_4H$, 424.17147; found 424.17054

m.p.: 229.5-231.2 °C

3.6 Synthesis of dendron precursor (6)

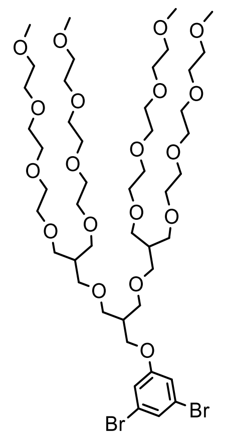

6

The procedure used for the synthesis of the dendron precursor **6** has been reported by Lee and co-workers.[1]

^{1}H NMR (400 MHz, $CDCl_3$) δ 7.22-7.13 (m, 1H), 7.06 – 6.88 (m, 2H), 3.97 (t, *J* = 5.4 Hz, 2H), 3.67 – 3.38 (m, 64H), 3.37 (d, *J* = 1.5 Hz, 13H), 2.32 (dd, *J* = 7.4, 4.0 Hz, 1H), 2.21 – 2.09 (m, 2H).

HRMS-ESI Orbitrap (m/z): [$M+H^+$] calculated for $C_{46}H_{84}Br_2O_{19}H$, 1101.40259; found 1101.40437

3.7 Synthesis of VsA monomer

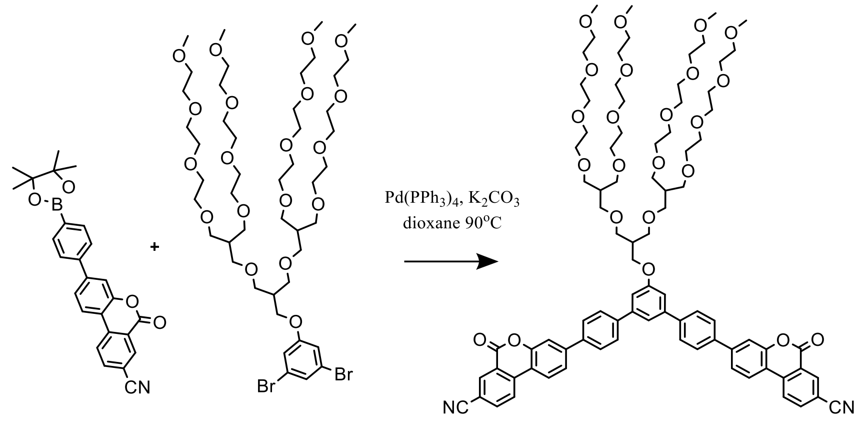

5 6 VsA

In a three neck flask equipped with reflux condenser was loaded with 25 mg of **5** (0.06 mmol, 3 eq.), 22 mg of **6** (0.02 mmol, 1 eq), $Pd(PPh_3)_4$ (2.5 mg, 10%), 2 mL of a 2M aqueous solution of K_2CO_3 and 5 mL of dioxane. The reaction mixture was stirred vigorously while degassing under nitrogen for 15 minutes. Next, the reaction mixture was kept under inert atmosphere, stirring for 30 hours at 90˚C. After checking product formation by TLC chromatography, the flask was cooled to room temperature, the two phases were separated and the aqueous phase was extracted with ethyl acetate. The combined organic layers were washed with water, dried over

[1] Kim, H.-J., Zin, W.-C. & Lee, M. Anion-directed self-assembly of coordination polymer into tunable secondary structure. *Journal of the American Chemical Society* **126,** 7009–7014 (2004).

Na_2SO_4, filtered and concentrated. The crude was purified by preparative TLC chromatography (silica, ethyl acetate/methanol 95:5). Yield: 9 mg (29%) of **VsA** as a pale yellow solid.

^{1}H NMR (400 MHz, $CDCl_3$) δ 8.72 (d, *J* = 1.8 Hz, 2H), 8.27 (d, *J* = 8.5 Hz, 2H), 8.16 (d, *J* = 8.4 Hz, 2H), 8.06 (dd, *J* = 8.4, 1.8 Hz, 2H), 7.85 – 7.75 (m, 8H), 7.75 – 7.69 (m, 4H), 7.49 (t, *J* = 1.5 Hz, 1H), 7.21 (d, *J* = 1.5 Hz, 2H), 4.17 (d, *J* = 5.7 Hz, 2H), 3.67 – 3.44 (m, 64H), 3.35 (s, 12H), 2.51 – 2.38 (m, 1H), 2.24 – 2.17 (m, 2H).

^{13}C NMR (101 MHz, $CDCl_3$) δ 159.37, 152.33, 145.62, 138.66, 138.22, 136.98, 135.12, 129.19, 128.93, 128.81, 127.87, 127.12, 127.07, 123.92, 122.88, 121.73, 117.42, 116.16, 115.37, 112.48.

HSQC

δ (ppm)

70.68	3.63
122.97	8.26
124.14	8.14
127.29	7.67
129.12	7.51
135.26	8.71
137.25	8.04

HRMS-ESI Orbitrap (m/z): [M+Na$^+$] calculated for C86H104N2O23Na, 1555.6920; found 1555.6922

UV-VIS: (CH_3CN) $\lambda_{max} = 284$ nm ($\log_{10}$ ε 4.5); $\lambda_{max} = 357$ nm ($\log_{10}$ ε 4.3)

FTIR: ν (CO) 1727 cm^{-1} (sh, s); ν (CN) 2233 cm^{-1}(sh, w)

m.p.: 149.4-151.2 °C
